# Vasculo-Protective Effects of Standardized Black Chokeberry Extracts in Mice Aorta

**DOI:** 10.3390/ijms252413520

**Published:** 2024-12-17

**Authors:** Valentina Buda, Adrian Sturza, Daliana Minda, Zorița Diaconeasa, Cristian Iuhas, Bianca Bădescu, Cristina-Adriana Dehelean, Corina Danciu, Mirela-Danina Muntean, Rodica Lighezan, Maria-Daniela Dănilă

**Affiliations:** 1 Faculty of Pharmacy, “Victor Babeș” University of Medicine and Pharmacy of Timișoara, E. Murgu Sq., No. 2, 300041 Timisoara, Romania; 2 Research Centre for Pharmaco-Toxicological Evaluation, “Victor Babeș” University of Medicine and Pharmacy of Timișoara, E. Murgu Sq., No. 2, 300041 Timisoara, Romania; 3 Department III Functional Sciences—Pathophysiology, Faculty of Medicine, “Victor Babeș” University of Medicine and Pharmacy of Timișoara, E. Murgu Sq., No. 2, 300041 Timisoara, Romania; 4 Centre for Translational Research and Systems Medicine, “Victor Babeș” University of Medicine and Pharmacy of Timișoara, E. Murgu Sq., No. 2, 300041 Timisoara, Romania; 5 Faculty of Food Science and Technology, University of Agricultural Science and Veterinary Medicine of Cluj-Napoca, 400372 Cluj-Napoca, Romania; 6 Faculty of Medicine, “Iuliu Hatieganu” University of Medicine and Pharmacy of Cluj-Napoca, 400012 Cluj-Napoca, Romania; 7 Department IV Biochemistry & Pharmacology—Pharmacology, Faculty of Medicine, “Victor Babeș” University of Medicine and Pharmacy of Timișoara, E. Murgu Sq., No. 2, 300041 Timisoara, Romania; 8 Department XIII Infectious Diseases—Parasitology, Faculty of Medicine, “Victor Babeș” University of Medicine and Pharmacy of Timișoara, E. Murgu Sq., No. 2, 300041 Timisoara, Romania

**Keywords:** black chokeberry, dried and frozen extracts, endothelial dysfunction, oxidative stress, superoxide anion, hydrogen peroxide, HLPC characterization

## Abstract

Black chokeberry (*Aronia melanocarpa* <Michx.> Elliot) represents a rich source of dietary polyphenols and other bioactive phytochemicals with pleiotropic beneficial cardiovascular effects. The present study was aimed at evaluating the ex vivo effects of two black chokeberry extracts (BChEs), obtained from either dry (DryAr) or frozen (FrozAr) berries, on oxidative stress and vascular function in mice aortic rings after incubation with angiotensin 2 (Ang 2), lipopolysaccharide (LPS) and glucose (GLUC) in order to mimic renin–angiotensin system activation, inflammation and hyperglycemia. The identification of phenolic compounds was performed by means of liquid chromatography with a diode array detector coupled with mass spectrometry using the electrospray ionization interface. The BChE obtained from the FrozAr was rich in cyanidin glucoside, rutin and caffeic acid, while the one obtained from the dried berries was rich in rutin, caffeic acid and chlorogenic acid. Mice aortas were dissected and acutely incubated (12 h) with Ang2 (100 nM), LPS (1 µg/mL) or GLUC (400 mg/dL) in the presence vs. absence of the two BChEs (1, 10, 50, 75, 100, 500 µg/mL). Subsequently, the tissues were used for the assessment of (i) hydrogen peroxide (H_2_O_2_) and superoxide production (using two methods, spectrophotometry and immunofluorescence), (ii) H_2_O_2_ scavenger effect and (iii) vascular reactivity (using the organ bath/myograph system). After exposure to Ang2, LPS or GLUC, both types of extracts decreased the H_2_O_2_ and superoxide levels in a concentration-dependent manner starting from either 50 µg/mL or 100 µg/mL. Also, in the highest concentrations (100 µg/mL, 150 µg/mL and 500 µg/mL), both extracts elicited a significant scavenger effect on H_2_O_2_ (similar to catalase, the classic H_2_O_2_ scavenger). Moreover, at 100 µg/mL, both extracts were able to significantly improve vascular relaxation in all stimulated aortic rings. In conclusion, in mice aortas, black chokeberry extracts in acute application elicited a concentration-dependent vasculo-protective effect through the reduction of oxidative stress and the alleviation of endothelial dysfunction in ex vivo conditions that mimic cardio-metabolic diseases.

## 1. Introduction

According to the last report on the burden of cardiovascular disease (CVD), global death counts due to CVD increased from 12.4 million in 1990 to 19.8 million in 2022 due to both population growth and aging, with Eastern Europe having the highest age-standardized mortality rates, mainly attributable to high systolic blood pressure [1]. Also, over the past two decades, the global prevalence of metabolic diseases has constantly risen and, more alarming, the mortality rates for diabetes and obesity has remained unchanged, being the highest in Eastern Mediterranean low-income countries [2].

Epidemiologic and experimental studies have unequivocally demonstrated that a high intake of dietary polyphenols may prevent CVD in the general population and reduce cardiovascular events in patients at risk of CVD [3,4] via mitigating oxidative stress, inflammation and vascular cell senescence, thus alleviating endothelial dysfunction [5,6] and improving cholesterol metabolism, thus preventing atherosclerosis [7], and, last but not least, modulating mitochondrial bioenergetics [8] and dynamics [9], ultimately providing cytoprotection.

Blueberries have a high phenolic content and have been extensively studied for their beneficial effects on various cell lines, animal models and human pathologies [10,11].

Black chokeberry (*Aronia melanocarpa* <Michx.> Elliot) is a member of the *Rosaceae* family and is a rich source of dietary polyphenols and other bioactive phytochemicals. Its origin belongs to the eastern parts of North America, where it was highly valued and used to cure colds [12]. In Europe, it was firstly recognized as an ornamental plant and was used as an anti-hypertensive drug in the 19th century [13,14]. Black chokeberry fruits have a sour taste and astringent characteristics, being, therefore, rarely used as fresh fruits [13]. Currently they are used in European countries for the large-scale production of juices, teas, wines, jams, liqueurs and schnapps or as food additives/preservatives [12].

Black chokeberry has a complex nutritional profile consisting of polyphenols (procyanidins, anthocyanidins, phenolic acids, flavonoids), minerals (K, Ca, Mg, Na, Fe, Zn, Cu, P), vitamins (such as A, B1, B2, B3, B5, B6, B9, C, E, K, amygdalin), carbohydrates, organic acids, amino acids, dietary fibers (celluloses, pectin, lignin and hemicelluloses) and more than 48 volatile compounds that contribute to its flavor (alcohols, aldehydes, esters, ketones and terpenes, e.g., limonene and β-caryophyllene) [12,15,16]. Procyanidins are the major class of polyphenolic compounds (>60% of total polyphenols). Their highest concentration has been observed in unripe fruits, followed by a decrease in concentration after the fruit development. Black chokeberry is particularly noteworthy for its high anthocyanin content, which represents approximately 25% of the total polyphenolic content. This makes black chokeberry one of the richest plants in anthocyanins (glycosylated cyanidins). The phenolic acids, including chlorogenic and neochlorogenic acids, make up 7.5% of the total polyphenolic content. Flavonols, such as quercetin, are present in a lower amount (approximately 1.3%), with a higher concentration found in the leaves rather than the fruits. Its composition is extremely variable, depending on many factors like climate conditions (warm and dry climate increase the total polyphenol content), soil composition, maturity of the berries, harvesting methods and storage conditions [13,15].

Regarding their complex phytochemical composition, the black chokeberry fruits have been systematically investigated during the past decades for their anti-oxidant, anti-inflammatory, anti-hypertensive, anti-atherosclerotic, anti-diabetic, anti-platelet, anti-microbial, anti-cancer and immunomodulatory properties in numerous experimental settings and also in humans with non-communicable diseases, mostly in cardio-metabolic pathologies and their comorbidities [12,15,16,17,18,19,20,21,22]. Last but not least, there is an increasing interest for developing supplements/functional foods with Aronia alone or in combination for synergistic activity in order to increase sport performance [23,24].

Oxidative stress is the common pathomechanism in cardiometabolic disorders and Aronia berries are recognized as a source of natural anti-oxidants, yet the number of studies assessing the direct effect of Aronia extracts on vascular reactivity and oxidative stress is rather limited.

The present study was double-aimed to (i) characterize the polyphenolic composition of two black chokeberry extracts (BChEs) obtained from dried and frozen Aronia fruits and (ii) assess their anti-oxidant effect and the catalase-like scavenger one as being responsible for the vasculo-protective effects in isolated mice aortic rings after incubation with angiotensin 2 (Ang2), lipopolysaccharide (LPS) and high glucose (GLUC) in order to mimic the classic neurohumoral changes (i.e., renin–angiotensin system activation, inflammation and hyperglycemia) commonly found in cardio-metabolic diseases.

## 2. Results

### 2.1. Polyphenolic Composition of the Extracts from the Frozen (FrozAr) and Dried (DryAr) Aronia Fruits

The chromatographic analysis of black chokeberry extracts revealed ten different phenolic compounds, such as cyanidin-3-*O*-diglucoside, neochlorogenic acid, cyanidin-3-*O*-glucoside, chlorogenic acid, cyanidin-3-*O*-arabinoside, cyanidin-3-*O*-xyloside, caffeic acid, rutin, quercetin-3-*O*-glucoside and quercetin, corresponding to a mix of phenolic compounds, such as anthocyanins, hydroxycinnamic acids and flavonols, as shown in Table 1; Table 2 (in the latter they are expressed as chlorogenic acid equivalents (CCEs)).

Quantitative analysis revealed that the extract obtained from the frozen black chokeberries (FrozAr) was rich in anthocyanin (Cy-3-*O*-glucoside, 7562.22 µg/g CCE), flavonol (rutin, 5989.25 µg/g CCE) and hydroxycinnamic acid (caffeic acid, 3752.98 µg/g CCE), while the extract obtained from the dried black chokeberries (DryAr) was rich in flavonols (rutin, 6136.61 µg/g CCE) and hydroxycinnamic acids (caffeic acid, 4224.30 µg/g CCE and chlorogenic acid, 3024.26 µg/g CCE), as shown in Table 2.

As depicted in Table 2, Cy-3-*O*-glucoside (7562.218 µg/g CCE) and rutin (6136.608 µg/g CCE) were the most abundant compounds in the two extracts, with the total phenolic content being higher for the FrozAr extract compared with the DryAr extract.

### 2.2. Black Chokeberry Extracts Reduced Oxidative Stress in Mice Aorta After Ex Vivo Stimulation with Angiotensin 2, Lipopolysaccharide and High Glucose

We tested the capability of increasing concentrations (1, 10, 50, 75, 100, 500 µg/mL) of dry (DryAr) and frozen (FrozAr) BChE to mitigate superoxide anion and hydrogen peroxide (H_2_O_2_) generation induced by acute ex vivo exposure of the mice aortic rings to Ang2 (100 nM), LPS (1 pM) or high GLUC (22.2 mM).

**Figure 1:**
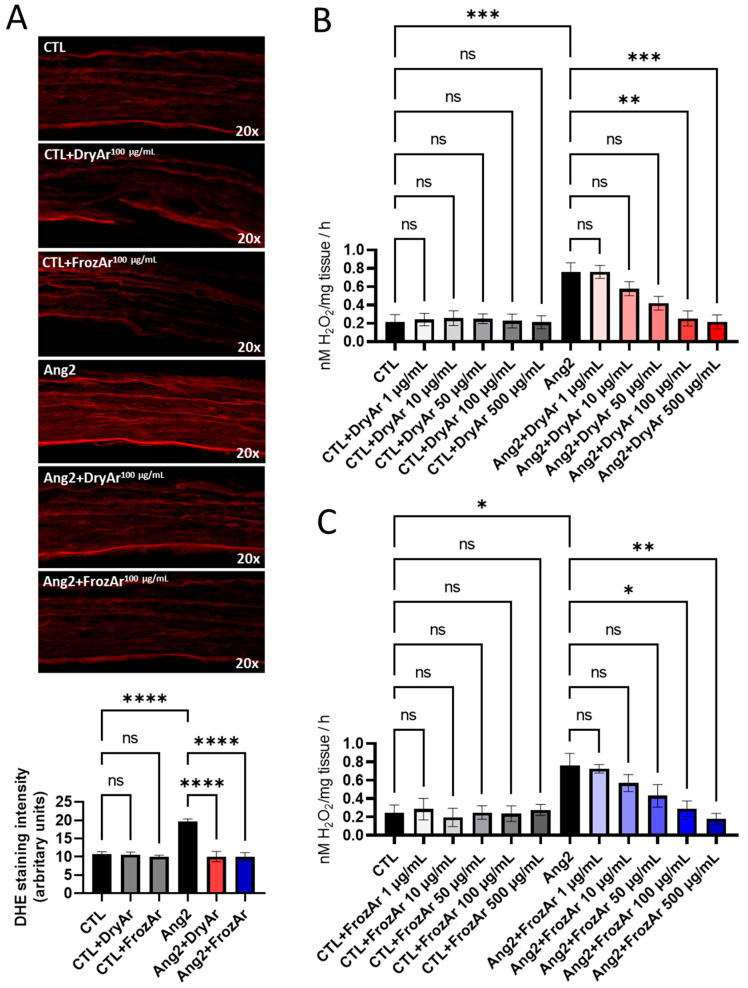
Effect of BChE on oxidative stress in mice aorta incubated (12 h) with Ang2 (100 nM). (**A**) DHE staining (one-way ANOVA, F = 37.69, *p* < 0.0001); (**B**) FOX assay using BChE obtained from dried berries (DryAr; one-way ANOVA, F = 7.643 *p* < 0.0001); (**C**) FOX assay using BChE obtained from frozen berries (FrozAr; one-way ANOVA, F = 4.909, *p* < 0.0001). Tukey test, ns = non-significant, * *p* < 0.05, ** *p* < 0.01, *** *p* < 0.001, **** *p* < 0.0001. n = 4.

**Figure 2:**
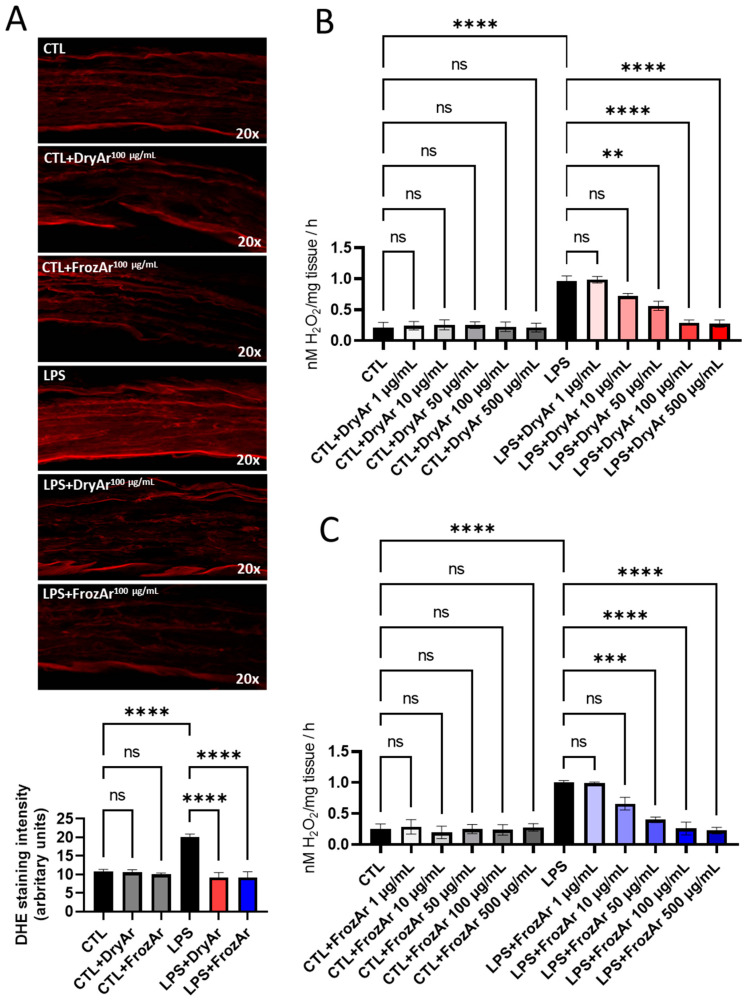
Effect of BChE on oxidative stress in mice aorta incubated (12 h) with LPS (1 pM). (**A**) DHE staining (one-way ANOVA, F = 42.43, *p* < 0.0001); (**B**) FOX assay using BChE obtained from dried berries (DryAr; one-way ANOVA, F = 20.23, *p* < 0.0001); (**C**) FOX assay using BChE obtained from frozen berries (FrozAr; one-way ANOVA, F = 14.21, *p* < 0.0001). Tukey test, ns = non-significant, ** *p* < 0.01, *** *p* < 0.001, **** *p* < 0.0001. n = 4.

**Figure 3:**
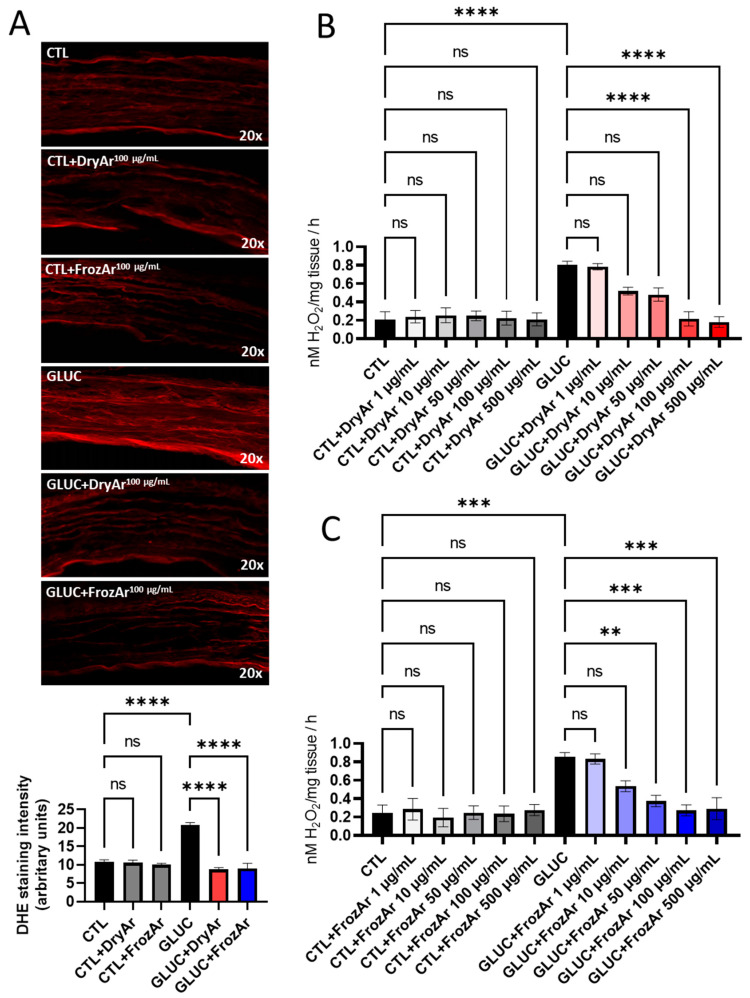
Effect of BChE on oxidative stress in mice aorta incubated (12 h) with high GLUC (22.2 mM). (**A**) DHE staining (one-way ANOVA, F = 57.54, *p* < 0.0001); (**B**) FOX assay using BChE obtained from dried berries (DryAr; one-way ANOVA, F = 12.12, *p* < 0.0001); (**C**) FOX assay using BChE obtained from frozen berries (FrozAr; one-way ANOVA, F = 8.217, *p* < 0.0001). Tukey test, ns = non-significnat, ** *p* < 0.01, *** *p* < 0.001, **** *p* < 0.0001. n = 4.

As revealed by dihydroethidium (DHE) staining (for superoxide anion) and the ferrous iron–xylenol orange oxidation (FOX) assay (for hydrogen peroxide), both types of extracts reduced their level in a concentration-dependent manner after exposure to Ang2 (Figure 1), LPS (Figure 2) or high GLUC (Figure 3).

Acute ex vivo exposure to Ang2 significantly increased H_2_O_2_ production, determined by the FOX assay in isolated murine aortic rings (Figure 1B, *p* < 0.001). Co-incubation with the DryAr extract elicited a concentration-dependent anti-oxidant effect that was evident from 10 µg/mL and reached statistical significance at 100 µg/mL (Figure 1B, *p* < 0.01) and 500 µg/mL (Figure 1B, *p* < 0.001). A similar significant effect on the Ang2-related oxidative stress was found for the FrozAr extract applied in the two higher concentrations, 100 µg/mL (Figure 1C, *p* < 0.05) and 500 µg/mL (Figure 1C, *p* < 0.01), respectively. No effect was reported when the control samples (non-incubated with Ang2) were exposed to the increasing concentrations of the extracts, demonstrating the lack of oxidative stress in the untreated isolated murine aortas.

We further assessed the effect of the 100 µg/mL concentration for both DryAr and FrozAr extracts on the superoxide anion production in immunofluorescence using the DHE probe in the vascular preparations stimulated or not with Ang2. We found the same powerful anti-oxidant effect of FrozAr and DryAr on the stimulated samples and no effect on the control (non-stimulated) ones (Figure 1A, *p* < 0.0001).

The same protocol was applied for assessing the inflammation-driven oxidative stress (in vascular samples incubated with LPS) and hyperglycemia-related one (in those incubated with high GLUC).

In the aortic rings acutely exposed to LPS, the concentration titration resulted in a significant anti-oxidant effect starting from 50 µg/mL for DryAr (Figure 2B, *p* < 0.01) and FrozAr (Figure 2C, *p* < 0.001). The two higher concentrations (100 µg/mL and 500 µg/mL) were more potent in decreasing the H_2_O_2_ production in the stimulated preparations and had no effect on the non-stimulated ones (FOX assay, Figure 2B,C, *p* < 0.0001). Similarly, with Ang2, acute co-exposure of the rings to LPS and either extract (100 µg/mL) significantly decreased superoxide generation in immunofluorescence (DHE stain, Figure 2A, *p* < 0.0001).

Last but not least, the vascular samples were acutely exposed to high glucose to mimic the pathological condition of uncompensated diabetes. The FrozAr extract (but not the DryAr one) already elicited a significant anti-oxidant effect at 50 µg/mL (Figure 3C, *p* < 0.01). When applied at the two highest concentrations, in the presence of high GLUC, both extracts significantly decreased the vascular level of H_2_O_2_ in the murine aortic rings (Figure 3B, *p* < 0.001 and Figure 3C, *p* < 0.0001) assessed with the FOX assay. A comparable significant anti-oxidant effect was obtained for the superoxide anion when the vascular samples were co-incubated with LPS and either the DryAr or the FrozAr extract at 100 µg/mL and analyzed in confocal microscopy with the DHE probe (Figure 3A, *p* < 0.0001).

### 2.3. Black Chokeberry Extracts Exhibited a Concentration-Dependent Catalase-like Scavenger Effect (H_2_O_2_ Neutralization)

**Figure 4:**
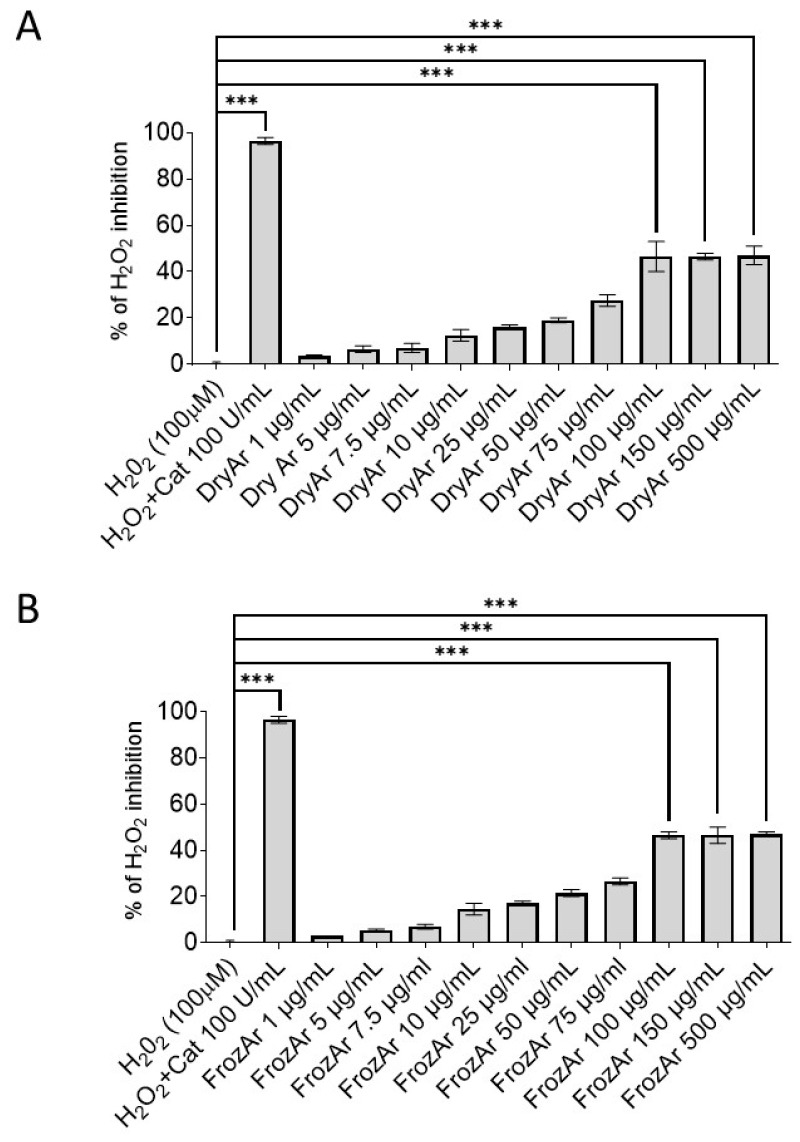
Catalase-like scavenger effect of BCh extracts. (**A**) Dried berries (one-way ANOVA, F = 109.9, *p* < 0.001); (**B**) frozen berries (one-way ANOVA, F = 296, *p* < 0.001) (experiments run in triplicate). Cat—catalase, Tukey test, *** *p* < 0.001.

In order to elucidate the mechanism underlying the anti-oxidant effect, we further assessed whether BChE exhibited a catalase-like scavenger effect and tested this by assessing the % of H_2_O_2_ (100 µM) neutralization by increasing concentrations of DryAr (Figure 4A) and FrozAr (Figure 4B) extracts. Catalase (Cat, 100 U/mL), the classic H_2_O_2_ scavenger, was used as the positive control. The results revealed that both extracts showed a significant (*p* < 0.001) inhibitory effect, starting from the concentration of 100 µg/mL (Figure 4A,B). This finding is in line with the above mentioned observations that both FrozAr and DryAr vascular H_2_O_2_ production when applied at the highest concentrations (100 and 500 µM) in the stimulated aortic samples and suggests that other indirect mechanisms contribute to their anti-oxidant effect (other than the direct scavenger one).

### 2.4. Black Chokeberry Extracts Improved Vascular Relaxation in Mice Aorta After Ex Vivo Stimulation with Ang2, LPS and GLUC

**Figure 5:**
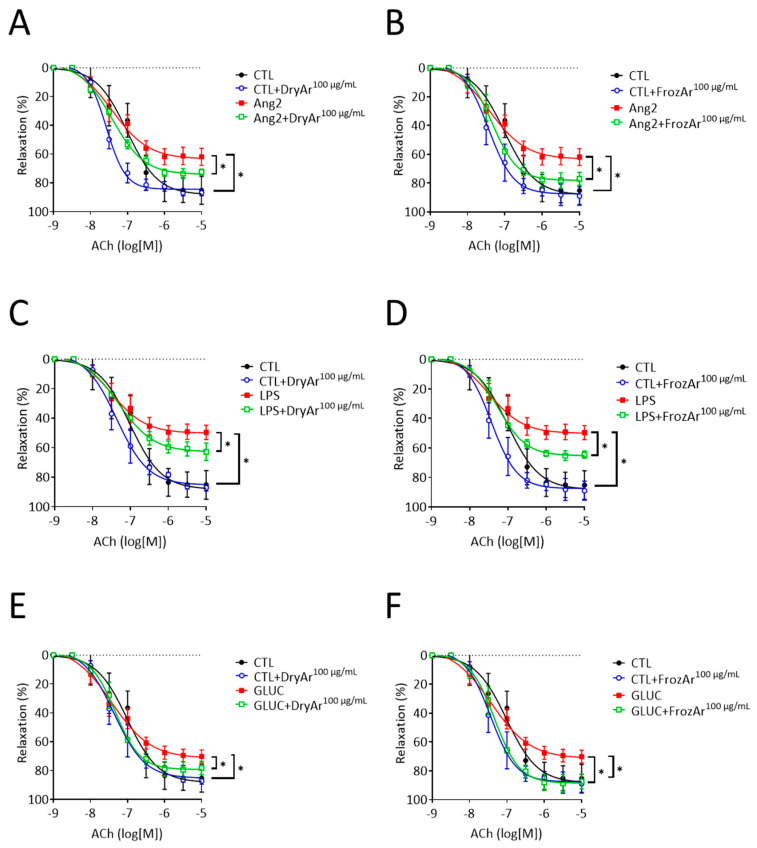
Effects of BChE (100 µg/mL) on vascular relaxation in mice aortas incubated or not with Ang2 (100 nM, 12 h), LPS (1 pM, 12 h) or GLUC (22.2 mM, 12 h). One-way ANOVA: (**A**) F = 8.487, *p* = 0.0027; (**B**) F = 4.891, *p* = 0.0190; (**C**) F = 15.51, *p* = 0.0002; (**D**) F = 14.59, *p* = 0.0003; (**E**) F = 3.491, *p* = 0.05; (**F**) F = 3.488, *p* = 0.05. Tukey test, * *p* < 0.05. n = 4.

The vascular effects of both extracts were further investigated using the concentration that elicited the constant significant anti-oxidant effect (100 µg/mL). As shown in Figure 5, incubation with Ang2 (A, B), LPS (C, D) and high GLUC (E, F) significantly impaired the endothelium-dependent relaxation to acethylcholine (Ach) of the murine aortic rings. Co-incubation with either type of BChE was able to partially restore the vascular relaxation of the stimulated samples (*p* < 0.05).

## 3. Discussion

The present study was aimed to characterize two BChE extracts prepared from dried and frozen Aronia fruits and assess their anti-oxidant and vasculo-protective effects.

The liquid chromatography coupled with diode array detection and electrospray ionization tandem mass spectrometry analysis showed that the FrozAr extract was rich in anthocyanins, flavonols and hydroxycinnamic acid (caffeic acid), while the DryAr extract was rich in flavonols and hydroxycinnamic acids (caffeic and chlorogenic acid). Our results are in accordance with the ones reported by Zielińska et al. These authors also showed that the chemical composition of black chokeberry extract changes during ripening and correlates with harvest time and cultivation region (e.g., temperature, altitude). As such, they reported a decrease in hydroxycinnamic acids associated with an increase in anthocyanin concentrations during seasonal growth and ripening, and high levels of anthocyanins specific for the ripped fruits [25].

We also found a total of phenolic content higher for the FrozAr extract compared with the DryAr one. This result is in line with the observation by Bushmeleva et al., who reported that drying at room temperatures reduced the total polyphenolic content (and elicited a higher preservation of flavonoids). Also, we found the highest antocyanin (Cy-3-O-glucoside) content in the extract obtained from frozen fruits, as reported by the same group [26]. Almost two decades ago, Pergola et al. reported that Cy-3-O-glucoside, the main anthocyanin from a blackberry extract, was the component responsible for its anti-inflammatory activity. Specifically, the compound inhibited the inducible nitric oxide synthase (iNOS) protein expression in the J774 macrophage cell line after stimulation with LPS, decreased the LPS-induced nuclear factor kappaB (NF- activation κB) and reduced extracellular signal-regulated kinase (ERK-1/2) phosphorylation [27]. In another study, performed on RAW 264.7 macrophage cell lines, Aronia extracts also inhibited Jun kinase (JNK) and p38 from the mitogen-activated protein kinase (MAPK) family (other than ERK) [28]. However, assessment of the anti-inflammatory activity of the extract was not the considered in the study. Nevertheless, the above mentioned signal transduction pathways may indirectly contribute to the oxidative stress mitigation.

Despite the variations in the phytochemical composition for the FrozAr and DryAr extracts, no major differences were seen regarding their anti-oxidant effects. We reported here that both extracts mitigated the vascular generation of the superoxide anion and hydrogen peroxide in ex vivo acute experimental conditions reported to elicit increased oxidative stress, namely incubation of the murine aortic rings with Ang2, LPS and high GLUC. The anti-oxidant effect was significant in all these settings for the two highest concentrations tested (100 µg/mL and 500 µg/mL) and was found for both hydrogen peroxide and superoxide anion. We further showed that the high concentrations (100 µg/mL, 150 µg/mL and 500 µg/mL) elicited a significant scavenger catalase-like effect, as a mechanism that partially explains their direct anti-oxidant property in this ex vivo experimental setting. However, a direct anti-oxidant effect of the blackberry juices, in particular the free radical quenching capacity estimated by the classic method of 2,2-diphenyl-1-picrylhydrazyl (DPPH) reduction, has been systematically reported in the literature (and reached approximately 90% in some studies [29]).

The anti-oxidant activity of black chokeberry has been extensively studied in the literature in order to provide a mechanistic approach for its use in preventing and/or treating numerous oxidative stress-related diseases [30]. The particularly high free radical scavenging capacity of chokeberry fruits has been confirmed over time by several groups that used different methods, such as oxygen radical absorbance capacity (ORAC), total reactive anti-oxidant potential (TRAP), ferric reducing anti-oxidant power (FRAP) and various radical scavenging assays, e.g., for hydroxyl radical, DPPH, nitric oxide (NO) radical [31].

Other than this direct effect, several other indirect mechanisms have been reported to contribute to the oxidative stress reduction by BChE, such as (among others) the decreased activity of pro-oxidant enzymes (e.g., xanthine oxidase, nicotinamide adenine dinucleotide phosphate hydrogen/NADPH oxidase), increased activity of anti-oxidant enzymes (e.g., catalase (CAT), superoxid dismutase (SOD), gluthatione peroxidase (GPx)), restoration of the reduced glutathione (GSH) concentration, chelation of transition metal ions (iron) and regulation of multiple signaling pathways (e.g., activation of the NRF2/Keap1 cytoprotective pathway) that modulate the intracellular redox status [32,33,34,35]. In the same vein, Rugina et al. investigated the effects of a chokeberry anthocyanin extract on high-glucose-induced oxidative stress in a pancreatic β-cell line (βTC3) and reported a reduction in intracellular reactive oxygen species (ROS) and an increased anti-oxidant capacity, i.e., higher levels of SOD, CAT, GPx and reduced glutathione (GSH) coupled with the insulin pool restoration [36].

The anti-oxidant potential of the black chokeberry was correlated in the literature with the total polyphenol content [37] and was ascribed mainly to the anthocyanin and proanthocyanidin fractions. An early study reported that cyanidin-3-arabinoside has been reported to possess the strongest radical-scavenging and inhibitory activity for 15-lipoxygenase and xanthine oxidase; it was also a potent α-glucosidase inhibitor, thus explaining the beneficial effects of Aronia extracts in reducing glycemia [38]. In an in vivo study in diabetic mice, Aronia juice reduced blood glucose level via the inhibition of dipeptidyl peptidase activity, the inhibitory effect being ascribed to the cyanidin 3,5-diglucoside fraction [39].

Gralec et al. conducted an interesting study aimed at measuring the anti-oxidant activity of Aronia melanocarpa during the fruit development and ripening period, using the ORAC and DPPH assays. They reported that the highest anti-oxidant activity was observed in unripe fruits; yet, currently, only the ripe berries are processed to obtain the extracts [40]. In a very recent study, the same group reported that the highest anti-oxidant properties of the unripe green chokeberry fruits could be attributed to the high content in chlorogenic acids [41]. We have to acknowledge as a limitation of this study that we did not assess the anti-oxidant property in relation to the activity of specific compounds/individual polyphenols.

Last but not least, another important finding of this study with respect to the anti-oxidant effect is that, in control vascular samples (non-incubated with Ang2, LPS or high GLUC), no effect on the H_2_O_2_ and superoxide levels was found for either dry or frozen BChE, regardless of the concentration applied. This observation is in line with the results published by Ryszawa et al., who investigated the effects of polyphenol-rich extracts of Aronia melanocarpa berries on superoxide production in platelets isolated from patients with cardiovascular risk factors vs. the control group. These authors reported a significant concentration-dependent superoxide decrease only in the diseased but not in the control group [42]. This observation is important because low physiological levels of ROS are key signaling molecules in a plethora of cellular processes, in line with the concept of oxidative eustress [43].

The present study also demonstrated the vasculo-protective effects of the BChE, which alleviated the endothelial dysfunction in isolated murine aortas stimulated by Ang2, LPS or high GLUC. Both FrozAr and DryAr extracts improved the endothelial-dependent relaxation to cumulative doses of Ach in experimental conditions that mimicked the internal milieu of patients with cardio-metabolic pathologies. Specifically, for these experiments, we used the concentration that elicited the significant anti-oxidant effect (100 µg/mL) for either extract. As such, the most severe impairment of the aortic rings relaxation was elicited by incubation with LPS, followed by Ang2 and high glucose. In the presence of either FrozAr and DryAr extracts, relaxation of the stimulated rings was significantly increased. Also, the BChE did not interfere with the relaxation of the control samples for the maximal cumulative dose of Ach. Nevertheless, the direct vascular protection elicited by the concentration that showed the anti-oxidant effect, demonstrated here in young animals, has to be recapitulated in experimental models of cardiometabolic pathologies and in old animals.

The results are in line with the pioneering study by Bell and Gochnaur, who investigated back to 2006 the vasoactive and vasoprotective properties of an anthocyanin-enhanced chokeberry extract (0.05 mg total anthocyanin per liter) on isolated porcine coronary arterial rings and showed the greatest protection against the loss of A23187—induced relaxation (the compound is a direct receptor-independent activator of endothelial constitutive NO synthase) following exposure to ROS from pyrogallol, when compared with bilberry and elderberry extracts. Moreover, these authors suggested that the direct vasoactive and vasoprotective effects could be attributed to the high anti- oxidant activity of the extract, which would reduce the concentration of ROS effectively reaching the arterial endothelium [44].

The group of Schini-Kerth later reported a potent endothelium-dependent relaxation elicited by Aronia juice in porcine coronary artery rings mediated by increased NO production and further tackled the underlying signal transduction of the vasoprotective effect in cultured endothelial cells. They reported the endothelial NO synthase (eNOS) phosphorylation via the redox-sensitive activation of the Src/PI3-kinase/Akt pathway and ascribed these effects to the conjugated cyanidins and chlorogenic acid fractions of the juice [45]. Very recently, an elegant study from the same group found that Aronia juice-triggered increased eNOS phosphorylation in cultured endothelial cells, which led to sustained NO production for 15 h. They further described that the redox-mediated activation of the phosphatidylinositol (PI) PI3-kinase/Akt, JNK and p38 MAPK pathways resulted in phophorylation and inactivation of the transcription factors FoxO1 and FoxO3a, thus relieving their repression on the eNOS expression.

Of note, Varela et al. also reported that a low concentration (0.1 μg/mL) of an Aronia extract applied on bovine coronary artery endothelial cells significantly induced NO synthesis and eNOS phosphorylation already after 10 min of incubation and the effects were further increased at 48 h [46].

Further studies on human samples are required in order to assess the concentration-dependent effect of the Aronia extracts, since it has been reported in the literature that high concentrations are pro-oxidant, i.e., increased lipid peroxidation in cell cultures [47]. Ultimately, while polyphenols display unequivocal beneficial effects in reducing oxidative stress and improving vascular function, we have to bear in mind that they are synthesized by plants in small amounts in order to provide defence against pathogens or adverse climatic conditions; conversely, when available in high amounts, they are deleterious, in line with the classic concept of hormesis (the biphasic dose–response, i.e., stimulatory in low dose, inhibitory in high dose) [48].

We have also to acknowledge as a limitation of this study the fact that only male animals were used for the experiments. While berry polyphenols target several signaling pathways, reducing oxidative stress, inflammation, apoptosis and cardiovascular remodeling, all relevant for the treatment and/or prevention of CVD in both sexes, sex-specific differences have been reported in the literature (reviewed in ref. [49]). The sex-specific beneficial effects of polyphenols (whole fraction and individual components) have been described in a plethora of experimental animal and cellular models, which obviously allow the mechanistic understanding of protection (albeit most of these studies were not explicitly designed to investigate the sex differences). As such, cardiovascular protection has been ascribed to the estrogen receptor-binding/signaling of polyphenols (other than their beneficial estrogen receptor-independent effects) and the phytoestrogen activity (mimicking the effects of endogenous estrogen, 17-estradiol) of the non-isoflavone polyphenols (e.g., quercetin, epicatechin, ellagic acid), respectively. Since the estrogen receptor expression is upregulated by the level 17beta-estradiol (E2), the main endogenous estrogen, the protective effects of polyphenols should be more evident in premenopausal females. However, mixed experimental findings were reported in the literature, with polyphenols eliciting either estrogenic cellular responses (comparable to those of E2) or antiestrogenic ones (by competing with E2 for the estrogen receptor binding sites, and thus, limiting its effects) [49]. Further specific investigations in humans are required to elucidate the sex-related differences of the polyphenol effects.

## 4. Materials and Methods

### 4.1. Polyphenolic Extract Preparation and Characterization

The plant material of *Aronia melanocarpa* (Michx.) Elliot was obtained from Aronia Charlottenburg Company in Timiș County, Romania, and was represented by the species’ fruits. Dried (at room temperature, approx. 23 °C, for a week) and frozen black chokeberries (fresh fruits refrigerated at −20 °C) were prepared at the Chair of Pharmacognosy of the Faculty of Pharmacy from “Victor Babeș” University of Medicine and Pharmacy of Timișoara, and were attributed to voucher specimen No. AM2/2022.

The plant material (5 g) was shredded and mixed with 10 mL of acidified methanol with HCl (0.3% *v*/*v*). The mixture was then subjected to ultrasound for 20 min, at 35 °C, 40 kHz, by means of an ultrasonic bath. The mixture obtained was then centrifuged (320 Hettich centrifuge, Tuttlingen, Germany) at 5000 rpm (rotational radius of 10.5 cm, relative centrifugal force of 2935) for 5 min and the supernatant was collected. Further, another 10 mL of solvent was added and centrifuged again. The procedure was repeated until the samples became colorless. The collected supernatant was subsequently filtered to vacuum and concentrated to dryness by rotary evaporation at 35 °C. The concentrated extract obtained was left in the oven, at 35 °C, for 4 days. The extract was stored at −20 °C until use.

Black chokeberry extracts were characterized at the Faculty of Food Science and Technology, University of Agricultural Science and Veterinary Medicine from Cluj-Napoca, Romania using the Agilent 1200 HPLC system equipped with a quaternary pump, solvent degasser, autosampler and UV-Vis detector with photodiode (DAD) coupled with mass spectrometry (MS) detector single quadrupole Agilent model 6110 (Agilent Technologies, Santa Clara, CA, USA). The separation of the compounds was performed on an Eclipse XDB C18 column, dimensions 4.6 × 150 mm, with 5 μm particles (Agilent Technologies, Santa Clara, CA, USA), using mobile phases A and B in the gradient below, for 30 min, at a temperature of 250 °C, with a flow rate of 0.5 mL/min. (Solvent A: water + 0.1% acetic acid; Solvent B: acetonitrile + 0.1% acetic acid; gradient (expressed in % B): 0 min, 5% B; 0–2 min, 5% B; 2–18 min, 5–40% B; 18–20 min, 40–90% B; 20–24 min, 90% B; 24–25 min, 90–5% B; 25–30 min, 5% B). The spectral values were recorded in the 200–600 nm range for all peaks. The chromatograms were recorded at the wavelength λ = 280, 340, 520 nm. For MS, the ESI positive ionization mode was used in the following working conditions: capillary voltage: 3000 V; temperature: 3000 °C; nitrogen flow: 8 L/min; m/z: 100–1200, full-scan. Data acquisition and interpretation of results were performed using Agilent ChemStation software (version B.02.01 SR2) (Agilent Technologies, Santa Clara, CA, USA). Identification and peak assignments of anthocyanins were based on their retention times and ultraviolet–visible spectra compared with standards and published data. Quantitative analysis was performed using 3 different calibration curves. Phenolic acids were expressed as chlorogenic acid equivalents, flavonols were expressed as rutin equivalents, while anthocyanins were expressed cyanidin equivalents.

### 4.2. Organ Culture of the Vascular Preparations

Young male mice (BALB/c strain, 4 weeks, 25 g) were purchased from Cantacuzino Institute (code S001), Bucharest, Romania. All animals were housed under controlled conditions (constant temperature and humidity of 22.5 ± 2 °C and 55 + 5%, 12 h light/dark cycle), with access to food and water ad libitum. The experimental protocol was approved by the Commission for Research Ethics of “Victor Babeș” University for Medicine and Pharmacy of Timişoara. All experiments were performed at the Centre for Translational Research and Systems Medicine of the Faculty of Medicine from “Victor Babeș” University of Medicine and Pharmacy in Timișoara, Romania.

Mice aortas were dissected under sterile conditions, cleaned (perivascular fat and connective tissues were carefully removed), cut into 3–4 mm rings and incubated for 12 h at 37 °C in EBM (endothelial basal medium) culture medium containing 0.1% BSA (bovine serum albumin), in the presence or absence of BChE (dry (DryAr) and frozen (FrozAr); 1, 10, 50, 75, 100, 500 µg/mL). In some experiments, the aortic rings were incubated with angiotensin 2 (Ang2—100 nM), lipopolysaccharide (LPS—1 μg/mL or 1 pM for a molecular weight of 1000 kDa) and high glucose (GLUC—400 mg/dL or 22.2 mM) in the presence vs. absence of BChE. Subsequently, the tissue was used for vascular reactivity experiments and measurements of hydrogen peroxide and superoxide production, respectively.

### 4.3. Assessment of Vascular Reactivity (Organ Bath Studies)

At the end of the 12 h organ culture, mice aortic rings were suspended in the organ baths of the myograph (DMT 620M) that contained 5 mL of Krebs–Henseleit solution (37 °C) and diclofenac (10 μM) to prevent the synthesis of the vasoactive prostaglandins, and aerated with 95% O_2_–5% CO_2_ gas mixture (pH 7.4). The rings were stretched to an optimal resting tension of 1 g and allowed to equilibrate for 30 min.

All protocols for the vascular reactivity assessment started with two consecutive contractions elicited by the addition to the organ bath of KCl (60 mM) in order to test for the integrity of the vascular media (the muscular layer). After reaching a stable plateau, the amplitude of contractile response was the reference for expressing the other various contractions. The integrity/function of the endothelial layer was further assessed by the ability of acetylcholine (Ach, 10 μM) to induce at least 10–15% relaxation of KCl-induced contraction. The rings were discarded if relaxation to Ach was absent or less than 10% of the KCl-induced contraction. Each experiment included control rings that were subjected to the same conditions, i.e., 12 h incubation in the culture medium (but without the drugs/extracts).

After assessing the presence of functional endothelium, the aortic rings were allowed to recuperate for at least 1 h, with the Krebs solution being replaced every 15 min, prior to the beginning of the vascular reactivity assessment protocol. Throughout the vasomotricity experiments, phenylephrine (Phe) was initially used for the preconstriction of the rings, followed by relaxation to cumulative concentrations of Ach.

A representative working tracing of the organ bath experiments is presented in the Supplemental Figure S1.

### 4.4. Assessment of Hydrogen Peroxide Production by Means of Spectrofotometry

Hydrogen peroxide generation was measured spectrophotometrically by means of ferrous iron–xylenol orange oxidation (FOX) assay, using the PeroxiDetect Kit (Sigma Aldrich-Merck, Darmstadt, Germany), previously described [50,51,52]. Hydrogen peroxide converts Fe^2+^ ions to Fe^3+^ under acidic conditions. The Fe^3+^ ions will form a compound colored with xylenol orange, which is spectrophotometrically quantified at 560 nm. H_2_O_2_ production is calculated from a standard curve. The result is expressed in nmol H_2_O_2_/hour/mg tissue. This protocol was performed on mouse aortic rings after 30 min incubation with angiotensin 2, lipopolysaccharide and glucose in the presence vs. absence of increasing concentrations of BChE (1, 10, 50, 75, 100, 500 µg/mL).

### 4.5. Assessment of Superoxide Production by Means of Immunofluorescence

Superoxide production was determined using the dihydroethidium (DHE) probe according to an immunofluorescence technique, as previously described [53,54]. Vascular samples were frozen in optimal cutting temperature (OCT) gel, cut in 20 μm sections and placed on the slide. After 3 consecutive washes with PBS (phosphate buffered saline, 3 times for 5 min), samples were incubated in the dark with DHE (30 min) at room temperature. The DHE in excess was removed by 3 washes with PBS. Subsequently, samples were mounted with Vectashield (Vector Laboratories, Berkeley, CA, USA) and visualized by confocal microscopy (Olympus Fluoview FV1000, Tokyo, Japan). The images were generated using laser excitation at 488 nm and emission at 610 nm in order to detect the superoxide specific product (2-OH-E+) according to ref. [55]. The images were further analyzed by means of Image J software (v. 1.52t, NIH, Bethesda, MD, USA), as described in ref. [56]. 

### 4.6. Assessment of the H_2_O_2_ Scavenger Catalase-like Effect of the Aronia Extracts

In order to dissect the anti-oxidant capacity of BChE, we tested whether it may act as an H_2_O_2_ scavenger. In this regard, dry and frozen BChE solutions (1, 5, 7.5, 10, 25, 50, 75, 100, 150, 500 μg/mL) were mixed with H_2_O_2_ solution (100 μM) and working color reagent (PeroxiDetect Kit, Sigma Aldrich-Merck) and incubated at room temperature (22–25 °C) for ~30 min. Catalase (100 U/mL, Sigma Aldrich-Merck) served as a positive control, being a classic H_2_O_2_ scavenger. The results were expressed as % of H_2_O_2_ inhibition.

### 4.7. Statistical Analysis

Statistical analysis was performed using GraphPad Prism software (v. 9.3.1, GraphPad, San Diego, CA, USA), with data being given as mean ± SEM. One-way ANOVA followed by Tukey’s post hoc test was used. The *p* values were corrected for multiple comparisons by the Tukey method. Data analysis of the concentration-effect response curves was performed using the ANOVA (comparisons of the bottom values, EC50 and the Hill slope; statistical significant data were obtained for the bottom values and no significant difference was found for the EC50 and Hill slope comparison). *p* values < 0.05 were considered statistically significant.

## 5. Conclusions and Future Outlook

We herein have characterized two Aronia extracts and demonstrated their vascular protective effect on isolated murine aortas via the mitigation of hydrogen peroxide and superoxide generation and improvement of the endothelial-dependent relaxation in ex vivo experimental conditions that mimicked the renin–angiotensin system activation, inflammation and hyperglycemia. However, future mechanistic studies are required to elucidate the contribution of the mechanisms underlying vasculo-protection, namely preventing the direct ROS-mediated damage to the endothelium and the chemical quenching of NO.

Whether these effects can be recapitulated in vivo in animal models of disease associated with endothelial dysfunction/oxidative stress and in human samples harvested from patients with cardio-metabolic pathologies is worth further investigation in order to provide a translational approach to the vascular protection afforded by various Aronia melanocarpa extracts and the future integration of the results into clinical practice.

## Figures and Tables

**Table 1:** Retention time (Rt), [M + H]^+^ and other MS ions, UV λmax of phenolic compounds identified in black chokeberry juice by LC-DAD-ESI-MS.

Peak No.	R_t_ (min)	[M + H]^+^ (m/z)	UV λ_max_ (nm)	Compound	Subclass
1	3.19	611, 449, 287	521, 280	Cy-3-*O*-diglucoside	Anthocyanin
2	9.88	355, 163	322	3-Caffeoylquinic acid (Neochlorogenic acid)	Hydroxycinnamic acid
3	10.98	449, 207	520, 280	Cy-3-*O*-glucoside	Anthocyanin
4	11.78	355, 163	322	5-Caffeoylquinic acid (Chlorogenic acid)	Hydroxycinnamic acid
5	11.96	419, 287	519, 279		Anthocyanin
6	12.13	419, 287	519, 279	Cy-3-*O*-xyloside	Anthocyanin
7	13.27	181, 163	320	Caffeic acid	Hydroxycinnamic acid
8	15.35	611, 303	355, 250	Q-3-*O*-rutinoside (Rutin)	Flavonol
9	16.16	465, 303	354, 250	Q-3-*O*-glucoside	Flavonol
10	21.71	303	356, 251	Q	Flavonol

LC-DAD-ESI-MS—liquid chromatography coupled with diode array detection and electrospray ionization tandem mass spectrometry; Cy—cyanidin; Q—quercetin.

**Table 2:** Qualitative and quantitative analysis of the phenolic compounds found in BChE, expressed as μg/g.

	FrozAr (µg/g CCE)	DryAr (µg/g CCE)	R_t_ (min)
Cy-3-*O*-diglucoside	1.39	0.42	3.19
Neochlorogenic acid	1624.59	1394.38	9.88
Cy-3-*O*-glucoside	7562.22	631.18	10.98
Chlorogenic acid	3056.90	3024.26	11.78
Cy-3-*O*-arabinoside	2105.20	200.02	11.96
Cy-3-*O*-xyloside	441.12	66.63	12.13
Caffeic acid	3752.98	4224.30	13.27
Q-3-*O*-rutinoside (Rutin)	5989.25	6136.61	15.35
Q-3-*O*-glucoside	1946.79	2985.63	16.16
Q	236.39	562.44	21.71
Total phenolic load	26,716.83	19,225.87	----

DryAr—extract obtained from dried black chokeberries; FrozAr—extract obtained from frozen black chokeberries. Phenolic acids are expressed as chlorogenic acid equivalents (CCEs), flavonols are expressed as rutin equivalents, while anthocyanins are expressed cyanidin equivalents.

## Data Availability

Data are contained within the article.

## Supplementary Materials

The following supporting information can be downloaded at: https://www.mdpi.com/article/10.3390/ijms252413520/s1.
