# Supplementary figures and images for: Vasculo-Protective Effects of Standardized Black Chokeberry Extracts in Mice Aorta
Source: Int J Mol Sci. 2024 Dec 17;25(24):13520. doi: 10.3390/ijms252413520 (PMC11677783; doi:10.3390/ijms252413520)

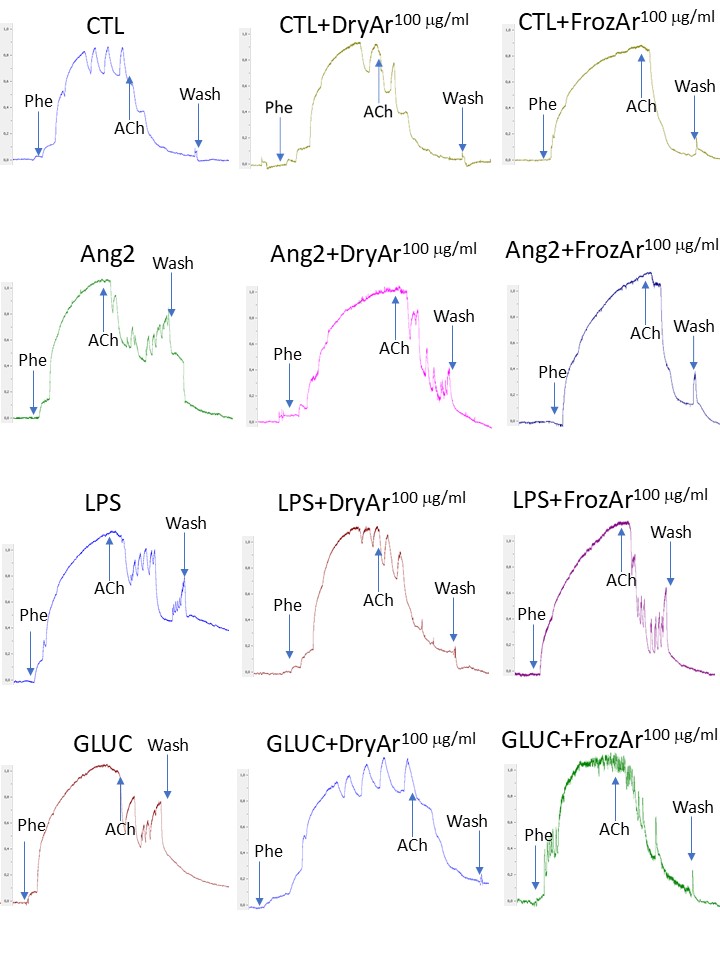

Supplement: Supplementary file 1 [file ijms-25-13520-s001.zip › ijms-3332945-supplementary.JPG]
